# Loneliness and disability: A systematic review of loneliness conceptualization and intervention strategies

**DOI:** 10.3389/fpsyg.2022.1040651

**Published:** 2023-01-25

**Authors:** Beni Gómez-Zúñiga, Modesta Pousada, Manuel Armayones

**Affiliations:** ^1^Psychology and Educational Sciences Studies, Universitat Oberta de Catalunya, Barcelona, Spain; ^2^eHealth Center, Universitat Oberta de Catalunya, Barcelona, Spain

**Keywords:** disability, loneliness, systematic review, intervention strategies, personal autonomy, accessibility

## Abstract

**Introduction:**

People with disabilities experience loneliness to a greater extent than people without disabilities. To better understand this problem, we have conducted a systematic review of studies that involved disability and loneliness. The aims are to research what loneliness is and to conceptualize and define it in the context of disability, and the intervention strategies that have been developed.

**Methods:**

The research protocol is based on the PRISMA guidelines. Two hundred and eighty-one papers were screened and 75 reports were assessed for eligibility.

**Results:**

We have not found whether loneliness in disability is a single construct or a collection of various subtypes. We have found that there are protective factors against loneliness in disabled people, such as having a job or living in an environment without physical barriers.

**Discussion:**

In terms of the interventions for people with disabilities, the same strategies have been adopted as for the non-disabled: social skills training, enhanced social support, opportunities for interactions, and cognitive training.

## Introduction

Disability is a global public health issue that affects one in seven people worldwide. In addition, everybody is likely to experience disability at some point in life, the World Health Organization says (World Health Organization, 2020). The same source adds that over a billion people live with a disability (93 million children and 720 million adults with significant functional difficulties). Moreover, due to an aging population and the increase of chronic health conditions, the number of people with disabilities is steadily increasing.

At the European level, the Ministry of Social Rights and the 2030 Agenda of the Government of Spain, and, specifically, the State Observatory of Disability (OED), issued the Informe Olivenza report (Jiménez, 2019) which compiled, systematized, updated, generated, and disseminated information on the subject of disability. According to this report, it is estimated that some 107 million people over the age of 16 in the European Union experience limitations in their activities, and around 32.5 million experience severe limitations. The highest prevalence is found in Latvia (40.1%), Estonia (39.7%), Slovenia (35.5), Finland (34.3%), and Austria (34.1%), and the lowest in Malta (11.9%) and Sweden (12.8%).

The same report states that the European Health and Social Integration Survey (EUROSTAT, 2019) considers a person to be disabled when he or she affirms that their health condition is a barrier to participation in any of the following areas: mobility to leave home, use of public or private transport, accessibility to buildings, access to training, access to suitable employment, using Internet, social contact with relatives, participation in leisure and cultural activities and problems paying for essential aspects of daily life.

According to the aggregate data, the prevalence of disability, in the terms thus defined, was 17.6% in the group of 26 countries belonging to the European Union that participated in the survey.

In addition, it is important to note that the prevalence of different types of disability changes with age. Disabilities related to communication, learning, the application of knowledge and task development, and those related to personal interactions and relationships are high among younger people with disabilities. Whereas, this type of disability decreases significantly with age, contrary to what happens with vision, hearing, mobility, self-care, and domestic-life disabilities, whose relative occurrence among people with disabilities increases over the years (Jiménez, 2019).

Alongside these data, the studies also allow us to affirm that people with disabilities, in addition to all the above problems, experience loneliness to a greater extent than people without disabilities (Emerson et al., 2021). For example, in Spain, 16% of non-disabled people live alone (Observatorio Estatal de la Discapacidad, 2019). Whereas, according to the study “La Soledad en España” (Díez and Morenos, 2015), more than 20% of people with disabilities live alone—and 38% of these people do so because they have to, not because they want to. Although living alone does not equate to loneliness, we may expect a higher likelihood of people with disability experiencing loneliness, due to their living situation, compared to the non-disabled population. In line with that, the perception of loneliness becomes a key factor in disability since it affects the quality of life of these people and their social integration (Emerson et al., 2021).

According to the Jiménez (2019), this loneliness is explained by various factors: accessibility, activity status (either having a job or participating in some kind of training activity), living environment, and fragility of support networks.

In order to better understand this problem, we have conducted a systematic review of different studies that involved disability and loneliness. The purpose of this systematic review is to establish the relationship between loneliness and disability and the intervention strategies that have been developed to counter loneliness among disabled people.

## Methods

This systematic review follows the Preferred Reporting Items of Systematic Reviews and Meta-Analyses (PRISMA) guidelines (Supplementary Appendix 1). A review protocol has been registered in the international prospective register for systematic review (PROSPERO; 2021 CRD42021270742, https://www.crd.york.ac.uk/PROSPERO/display_record.php?RecordID=270742).

### Search strategy

The literature search was conducted in March 2021 by a librarian, following the search strategy authors had previously cited (PROSPERO; 2021 CRD42021270742). The search strategy followed this syntax: “loneliness AND disability,” “loneliness AND impairment,” “loneliness AND disabled,” “solitude AND disability,” “solitude AND impairment,” and “solitude AND disabled.” Electronic databases searched include PsycINFO, PubMed, Scopus, and Web of Science.

### Inclusion and exclusion criteria

Studies were included if: (a) research focused on individuals with disability; (b) sample included participants of any age; (c) participants with disability and loneliness; (d) published between 2000 and 2021; (e) peer-reviewed; and (f) written in English or Spanish.

Studies were excluded if they were a journal, only the abstract, a letter or a review.

### Study selection

Two of the authors (BG-Z and MP) screened titles and abstracts of papers independently to identify relevant articles applying the inclusion and exclusion criteria. Items were excluded based on the title and abstract. Accordingly, irrelevant articles were removed. If it was not clear whether the study should be included, the full text of the study was retrieved, and the inclusion and exclusion criteria reapplied. Disagreements were resolved through consensus and discussion with a third reviewer if necessary (MA).

### Data extraction

A data extraction form/table was developed to include the aims of this systematic review. This table is based on the factors that explain loneliness (Jiménez, 2019) as they are detailed in the Section “Introduction”; in addition to these factors, we added key information about: authorship, year of publication, title, focus on loneliness and disability, sample (N and age), conceptualization of loneliness, type of disability and area of knowledge.

Data were extracted by two researchers (BG-Z and MP) and an assistant researcher.

## Results

**FIGURE 1:**
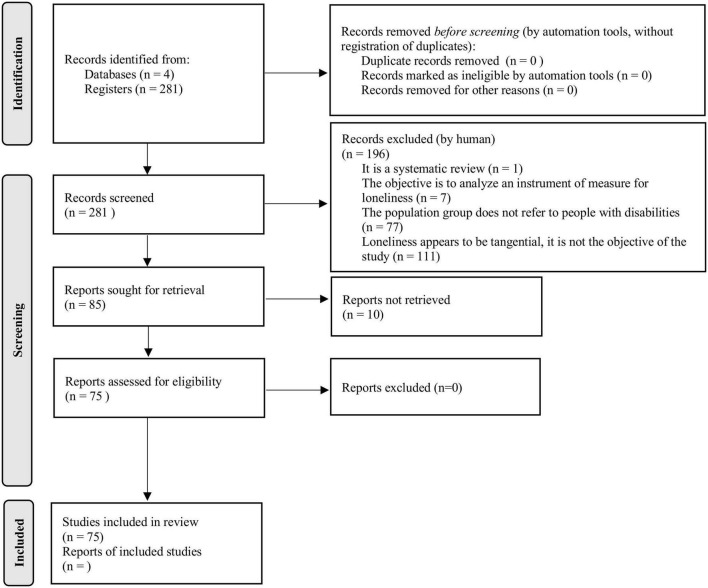
Flow chart of identification, screening, and inclusion process. Duplicates = 0 because the database search tools automatically remove them.

Figure 1 shows a flow chart of the selection process. We identified 281 studies from the database that met the inclusion criteria. One hundred and ninety-six non-relevant articles were excluded.

**FIGURE 2:**
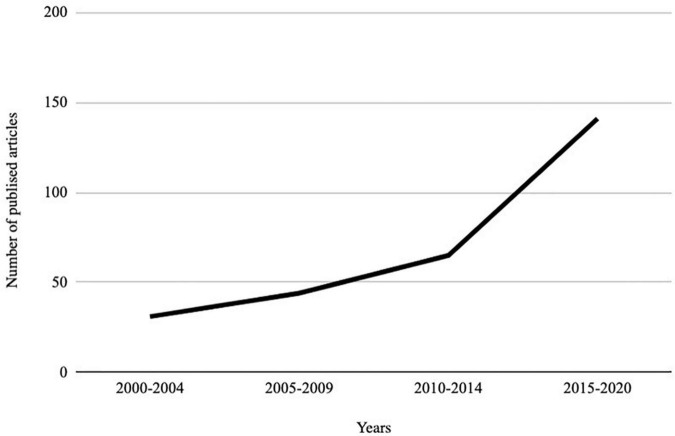
Evolution of the publication of articles on loneliness and disability from 2000.

We retained 281 articles for full-text evaluation and selected 75 articles for inclusion based on eligibility criteria. Figure 2 shows the change over time of the publication of the articles.

### Loneliness: Conceptualization

Of the total number of articles selected (75), only 27 were focused on loneliness as the main variable. Thus, to describe the concept of loneliness, we have taken into account only these 27 articles.

To begin with, we should point out that the authors who have conceptualized loneliness share the belief that social isolation and loneliness are not the same construct. While the first refers to an objective analysis of the network of social relationships that a person maintains, loneliness would be a subjective, unpleasant and, therefore, unwanted feeling that emerges when the quantity and intensity of these social relationships do not correspond with what is expected, with what is desired. From this cognitive perspective, the feeling of loneliness would imply, on the one hand, taking into account the history of one’s own relationships and, on the other, observing and evaluating other people’s relationships.

**FIGURE 3:**
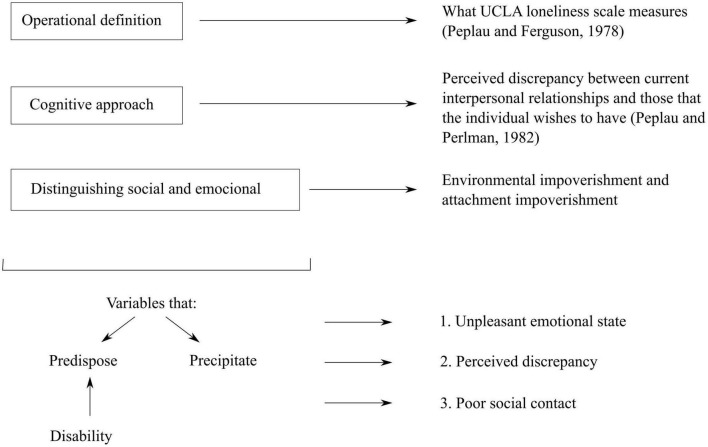
Diagram on the concept of loneliness.

In Figure 3 we provide a diagram on the concept of loneliness in the articles analyzed.

Having read the selected articles, we have found that the definitions of loneliness revolve around three main perspectives.

#### Operational definition

Scientists describe the operations or procedures that define the concept. Of the total analyzed articles that conceptualized loneliness, 12 articles defined loneliness operationally, and of these 12 articles, 5 used the UCLA scale. The other 7 articles used the De Jong Gierveld Loneliness Scale (2 articles), the Children’s Loneliness and Social Dissatisfaction Scale (2 articles), the Loneliness and Aloneness Scale for Children and Adolescents (LACA) (2 articles), and the Worker Loneliness Questionnaire (WLQ) (1 article).

#### Cognitive approach

Peplau and Perlman (1982) introduced this perspective, defining loneliness as the perceived discrepancy between current interpersonal relationships and those that the individual wishes to have. Thus, the perception of loneliness does not depend on an absolute number of interpersonal relationships, but is due to the possible discrepancy between the actual number of relationships and those that we would like to have.

#### Distinguishing social and emotional

It is common among authors to distinguish between social loneliness, associated with a poor network of contacts or insufficient social integration, and emotional loneliness, in which the determining element is the lack or loss of an essential attachment figure at an intimate level. In this second case, loneliness would not be resolved simply by increasing the number of contacts or relationship networks, but would require reestablishing or forging a relevant, significant bond with other people.

These three approaches when conceptualizing loneliness propose variables that predispose or precipitate the perception of loneliness. We have grouped the ones that predispose into three main variables: unpleasant emotional state, perceived discrepancy (as described above), and poor social contact. These variables lead an individual toward loneliness, in the sense that if they perceive some of these variables, it is more likely that they also perceive loneliness. Thus, these variables would be present before loneliness onset and predispose to its appearance. In addition, as a predisposing factor, the selected articles propose disability. In this sense, these articles present disability as a variable that would precede loneliness and that would cause the perception of loneliness almost inevitably. Understood in this way, disability would be a fundamental variable that would explain a large part of the perception of loneliness, with a direct relationship between disability and perceived loneliness.

### Sample used in the studies

The sample size used in the studies analyzed is extremely varied. Sample sizes ranged widely from 5 to 13,828 participants (M = 790.36, SD = 2.037). As such, we have located several studies with very small sample sizes; for example, the study by Stacey and Edwards (2013), with a sample size of five adults who have learning difficulties; the ones by Cooper et al. (2009), and by Ballin and Balandin (2007), who work, respectively, with six and seven adults affected by cerebral palsy; or some others with than 15 participants.

In contrast, other studies have compiled and analyzed data on a much larger scale. Several of them have worked with samples of more than a thousand people, but the following, in particular stand out: the research by Stickley et al. (2017), with a sample of 7,403 people affected by some type of mental illness; Stancliffe et al. (2010), with 7,996 adults who have developmental and intellectual disabilities; and Vitman Schorr and Khalaila (2018), who work with a sample of 13,828 people over age 65 who are experiencing functional difficulties.

In the cases of small sample sizes, there is not always a similar number of men and women. In particular, we have found 15 studies that reported the subjects’ gender; in nine of them, the number of males participants is larger than that of females. Only in one study the number of male and females was the same. This is fairly balanced in studies with larger samples.

Regarding the age of the participants, and considering those studies in which data is provided, we notice that studies focused on adults in a wide age range (from 18 years up) predominate; there is a total of 34 of these works. A group of 27 works centered around studying loneliness in children, adolescents, or young people under age 25. And in another smaller group are the 8 works that study loneliness in the population over 65 years of age (see Supplementary Appendix 1 for more details).

### Type of disability included in the studies

In relation to the type of disability included in the analyzed studies, they have been classified into the following categories, according to the criteria of the Department of Social Rights of the Generalitat de Catalunya (Spain) (Department of Social Rights, 2019): visual impairment, hearing impairment (also including communication disorders caused by various pathologies, including voice problems, throat diseases, or neurological diseases), physical disability (both motor and non-motor), intellectual disability, cognitive impairment or disorders of development, and mental disorders.

Based on this classification, we can say that only one study has focused exclusively on people with visual impairments, and only two more have focused on people with hearing or communication problems. In contrast, the studies that have analyzed the problems of loneliness in people with intellectual disabilities or developmental disorders are the most abundant (a total of 33), followed by those that focus on people with physical disabilities (20 works) and those that have included various types of disability (a total of 11 papers). It is worth noting that in one of the works, the only criterion used was age, having considered that being an older person was sufficient to understand there is some sort of disability, no matter how small. Finally, 6 works studied the feelings of loneliness in people with some kind of mental disorder.

### Dimensions for analyzing loneliness among people with disabilities

As stated in the Section “Methods,” and in accordance with the categories of the Informe Jiménez (2019), we structured the analysis of the information based on four large dimensions: accessibility, employment status, physical context, and support networks. To these we added a fifth dimension that, due to its practical relevance, we considered necessary: intervention strategies.

#### Accessibility

Of the total number of articles selected for the systematic review, only six included this variable in their analysis. In particular, accessibility is considered by authors such as Olsen (2018), to be a variable closely linked to stressors, such as barriers to access to public transports, and social or cultural events, etc. Other authors take this variable into consideration indirectly, indicating the degree of mobility of people with disabilities with whom they have worked (Lowe et al., 2021); Cooper et al. (2009), or architectural adaptations for people with physical disabilities (Tzonichaki and Kleftaras, 2002). The studies by Macdonald et al. (2018) and Tough et al. (2018) go further and propose that loneliness and social isolation in disability are not due to a pathological explanation, but rather environmental barriers related to the accessibility of physical spaces or leisure activities. Lastly, we would like to highlight the article by Vitman Schorr and Khalaila (2018), which is based on data collected in the Survey of Health, Aging, and Retirement in Europe (SHARE Project, 2020) covering 15 countries. The authors concluded that the easier the accessibility to services and sites in the living area, the greater the quality of life in old age. Perceived easier access to services and sites has also been found to be associated with lower social isolation and feelings of loneliness.

#### Employment status

Employment has been considered a reason for social exclusion (Olsen, 2018). In turn, Gascon (2009) took into account employment status and loneliness to affirm in his work (with a sample of 55 people, 27 of whom work in a regular work environment) that the loneliness experienced by individuals working in a regular environment seemed to emerge from social dissatisfaction at work.

Other works have taken a group of students as a sample, and as such have been defined in terms of employment status (training activity) (Papoutsaki et al., 2013; Feldman et al., 2016) or a group of older people, in which case their employment status was that of retiree (Alpass and Neville, 2003). Other studies, such as Beal and Stuifbergen (2007), Dor and Savaya (2008), Chou and Chronister (2012), Asselt-Goverts et al. (2018), or Kruithof et al. (2018), have taken this variable only as descriptive of the sample, but without being a variable included as part of the research hypotheses. However, the article by Macdonald et al. (2018) goes a step further and states that 86.5% of disabled participants who had experienced loneliness and isolation reported barriers to employment.

In the study by Balto et al. (2019), whether one is working or not working plays an important role in their conclusions. Specifically, less than half of the participants with disabilities were employed, while more than 90% of the control subjects worked. However, when this variable was controlled, the differences on the loneliness questionnaires was no longer significant between the two groups. It is possible that employment status could be a protective variable for the perception of loneliness.

#### Environmental context

The environmental context is not a factor that has been taken into consideration in the 75 articles analyzed in their entirety. The study by Lowe et al. (2021) states that seven participants live in the city and four in rural areas, but this is simply presented as demographic data for the sample. The same is true of work by Burholt et al. (2017) or the work done by Pavri and Monda-Amaya (2001).

We have only found two studies about environmental context and perceived loneliness. One of them is by Papoutsaki et al. (2013): children with mild intellectual disability (MID) living in smaller towns reported fewer feelings of loneliness than those living in the capital. The other, Stancliffe et al. (2007), describes how adults with intellectual or developmental disabilities (ID/DD) live in increasingly small community settings, where the risk of loneliness may be greater. They examined self-reported loneliness among 1,002 individuals with ID/DD from 5 states in relation to community residence size, and they found that more loneliness was reported by residents of larger community living settings, but with a weak association.

#### Support nets

In general, the articles that are part of this review do not take the support-net variable as one for analysis. Now, to the extent that perceived loneliness is very often related to social support, or support nets, many of the works analyzed take this factor into consideration, understood as traditional sources of support, whether from family, partners, friends, co-workers, peer group or support workers.

Overall, we would like to highlight some of the studies that we found particularly interesting.

In the article by Bigby et al. (2017), the authors focused on the growth of supported living or community places for housing. For their research, they took into account social connections, and it is within this context that they described the support-net variable as the informal support offered by family members and formal services. The people they worked with were adults with intellectual disabilities, and they positively valued the social contact with the family, with people close to the town where they lived, or the use of public or private facilities (gyms, pubs, etc.) to meet people.

Another study that took this variable into consideration was Bossaert et al. (2012). One of its main objectives was to explore the relations between number of friends and friendship quality, and loneliness in students with disabilities. The results showed that friendship quality did not play a significant role in predicting loneliness for typically developing students, students with ASD and students with motor and/or sensory disabilities, although there was a marginally significant effect of friendship quality on loneliness (−0.21, *p* < 0.05) for the entire group of students with SEN (special education needs). It seems that, unlike for typically developing students, the number of friends is not related to the prevalence of loneliness in students with ASD and students with motor and/or sensory disabilities.

Kruithof et al. (2018) worked with the support-net factor with the goal to enlarge networks and increase social participation. They found that an intervention that aims at network enlargement and increasing societal participation simply by getting people to come together as a group is not a sufficient strategy. They would need to implement instrumental support programs, such as social skills training and interventions to change activity patterns, since these programs have been found to be most successful in terms of network enlargement for people with mild intellectual disabilities (MID). This issue is related to the lack of social skills that may accompany some people with intellectual disabilities: when the social skills are not strong enough, they need to have prior training if that person is going to be placed in a situation interacting with others; if not, the interaction itself is not rewarding.

### Intervention strategies

Some of the works analyzed refer to potential intervention strategies that could be used by people with disabilities to alleviate their feelings of loneliness. Among these possible strategies cited by different authors, we highlight the following three, which would be like progressive steps to intervene on loneliness.

Firstly, the importance of income support in order to improve their opportunities for social participation and increase the quality of their lives (Rijken and Groenewegen, 2008). Thus, it would be very relevant when addressing loneliness for people with disabilities, to have a minimum amount of financial support.

Secondly, implementing social programs and policies concerned with adapting both the physical and social environment, to improve access to public services (transportation, bank, church, recreational center) and social roles (employment or voluntary work) (Hopps et al., 2001; Wormald et al., 2019). In other words, the policies of public and private organizations and entities should provide these people with both the physical and social environment that makes it possible to connect with others.

Thirdly, there would need to be programs concerned with learning and improving social skills and reducing anxiety in handicap situations (Hopps et al., 2003).

Notice that the first two strategies do not focus directly on the person who experiences loneliness, but rather they address the political, social, and contextual factors that favor it. Only the third strategy is directed toward the specific abilities or behaviors of people with disabilities.

As for specific information on programs that have been designed, implemented and, in some cases, evaluated to alleviate loneliness in people with disabilities, we have found certain guidelines that have to do with the type of disability being treated.

Hence, in the case of children with learning disabilities or SEN, the interventions we found focused on improving their ability to relate to others; through both programs that help them practice their skills at identifying and expressing their needs (Kotzer and Margalit, 2007), as well as interventions to improve interactions with children of their same age and gender and to strengthen their friendships (Bossaert et al., 2012; Papoutsaki et al., 2013).

In the case of adults with MID, we see that the proposals focus on developing programs that incorporate volunteers or mentors to promote social contacts. Thus, Kruithof et al. (2018) present a program to increase social relationships that is based on organizing a monthly community dinner, with about 20–30 participants and for the purpose of encouraging neighborhood participation and connection. A similar strategy is proposed by Fyffe and Raskin (2015) in their leisure buddy program, in which pairs are established between a person with a mild intellectual disability and a community volunteer, so that both meet regularly to do some sort of joint activity. Other works that also introduce this type of strategy are those by Maddox et al. (2017), Asselt-Goverts et al. (2018), or Wilson et al. (2020).

In the case of people with physical disabilities, Kalina et al. (2018) present the details of a study with an experimental design (RCT), which uses a 12-week educational program that works on self-management strategies. The results indicate that these strategies can improve self-efficacy among people with multiple sclerosis and that improved self-efficacy is associated with reduced feelings of loneliness. Also proven effective with this population group were cognitive and behavioral techniques based on an in-person individual assessment (which includes setting goals; reviewing knowledge and beliefs about social relationships, behavior, and emotions; reviewing social skills or reviewing self-presentation skills) (Hopps et al., 2003). In short, the strategies that are often used in the case of people with physical disabilities involve cognitive-behavioral programs to improve variables related to self-management and thoughts, beliefs and emotions.

When it comes to people who have suffered brain injury, Lowe et al. (2021) recommend starting psychotherapeutic processes. In the case of the 11 participants in their study, they work on psychoeducation, emotion management, and dialectal thinking. The results that they obtain are satisfactory, with the participants describing a desire and motivation to engage with the brain injury community and to volunteer or “give back.” This engagement with the brain injury community appeared to ease feelings of loneliness and increase openness, connectedness, and integration.

### Area of knowledge

We have followed the classification provided by the World Health Organization and World Bank (2011) for the six different contexts or areas from which disability can be analyzed and worked on: general health care, rehabilitation, assistance and support, enabling environments, education, and work and employment. Thus, in the summary table of the results, we incorporate a column that indicates which of these six areas each of the articles in the systematic review addresses as a priority.

Factoring in loneliness as a variable included in the analysis of our review already supposes a bias in the type of works found. For this reason, the most abundant are those that refer to education (a total of 22 articles on inclusive education, promoting learning skills for life, quality of education, etc.), to assistance and support elements (with a total of 19 articles supporting independent living, counseling services, etc.) and general health (18 articles); followed by the articles that deal with aspects of environmental and inclusive design (11 articles) and, finally, we find the works that focus on all the aspects of rehabilitation and on access and inclusion in the workplace (2 articles).

A summary of all the results can be found in Supplementary Appendix 2.

## Discussion

We have seen how certain aspects, such as some personal variables or social norms, could be considered predisposing factors to experiencing loneliness and along with them would be the precipitating factors, those directly involved in experiencing loneliness. The disability should be understood as one of the predisposing factors, as would the social skills that each one of us has or the behaviors or social habits that are established in our cultural surroundings. None of these elements determine on its own the onset of loneliness, but they affect the probability of it occurring.

One of the aims of this review has been to conceptualize loneliness in disability, but as we have seen in the previous Section “Loneliness: Conceptualization,” the work of the authors of the reviewed articles has not been entirely consistent. From our point of view, the concept is clearly multidimensional, but in the analyzed articles, we have not seen any further explanation of this issue at all.

In line with this concept of loneliness, scales have been designed so that it can be measured, the most common being the UCLA Scale from Russell et al. (1978) currently in its third version (UCLA-3). There are authors, however, who advocate the need for a qualitative, rather than quantitative, analysis of the subjective experience of loneliness, in order to understand it in its full dimension (Heinrich and Gullone, 2006; McVilly et al., 2006).

Most of the articles we have analyzed focus on a specific disability (stroke, intellectual disability, etc.), but do not analyze samples with a wide variety of conditions. For this reason, the data that we provide in this work may be seen as being somewhat fragmented or incomplete. However, given that the factors analyzed have been studied in samples that varied in terms of disability type, we believe that we can draw conclusions that could potentially apply to all disabled people, regardless of the specific type.

Regarding accessibility, although it is not a very frequently studied variable, the works that analyze it show a clear relationship between the environmental barriers that hinder mobility or access to an activity and an increase in stress, a decrease in quality of life, or the feeling of isolation. In this sense, we could conclude that reducing or eliminating this type of barrier (in transport, in access to public spaces and services or in leisure activities for all ages) is a particularly relevant intervention. In the New Zealand Disability strategy, the outcome number 5 is precisely that of Accessibility (Office for Disability Issues, 2022). Its perspective helps us to understand the impact of access to all places, services and information on disability needs.

It is also worth noting that this intervention depends entirely on public authorities and on the leaders of public and private businesses and organizations, and not on people with disabilities. We emphasize this aspect because often the responsibility for feeling lonely is placed on the one who experiences it, when certain measures that could clearly prevent it are beyond their reach and concern us all. Along these lines, concepts such as “universal design” which was coined by the European Institute for Design and Disability at their 2004 meeting (EIDD, 2014) and that many cities are developing can, indirectly, but also unequivocally, help reduce the perception of loneliness, by facilitating the active participation of people with disabilities in different social events, without physical or social barriers preventing them from doing so.

In terms of employment status, global data show that employment rates are lower for people with disability (for both men and women). Taking this into account, we were surprised to have found so few results that analyzed this variable when studying loneliness, and even fewer that considered it part of the intervention process to counter social isolation. Consequently, we believe that a specific analysis of work activity is necessary as a protective factor against loneliness in this group, examining the effect it has, depending on disability, or age, and also its potential differential role in the case of men and women. It is likely that this type of more specific study could benefit from a more qualitative than quantitative approach, precisely because of the wide range of variables to be considered and because of the very subjective dimension of the feeling of loneliness.

In their meta-analysis about interventions in loneliness, Masi et al. (2011) did not specifically focus on people with disabilities. However, we want to point out that they did discuss four fundamental types of intervention strategies: (a) improving social skills, (b) enhancing social support, (c) increasing opportunities for social contact, and (d) addressing maladaptive social cognition. The authors concluded that most successful interventions are those addressing maladaptive social cognition–that is, negative thoughts about self-worth and how other people perceive you–correcting them through cognitive behavior therapy, for instance. However, all of these strategies focus on the person experiencing loneliness, and moreover, there are no robust evaluations of the effects of community-based policy interventions that aim to improve the social environment and indirectly impact loneliness.

In the case of intervention strategies aimed specifically at people with disabilities who feel lonely, we have also observed that the programs focus on the individual, on how to help them, or give them tools to change that feeling of loneliness, regardless of any interventions at the collective or societal level. In addition, we have shown that the type of intervention varies according to the specific disability in question. Thus, training on social skills is the preferred strategy used when it comes to children or adolescents with learning difficulties; programs to increase the number of social contacts through volunteers or mentors are usually implemented for adult people with learning disabilities, while for loneliness associated with physical disability, intervention programs focus on training skills to improve the perception of self-efficacy.

Despite this certain coherence in the type of intervention, there are hardly any RCT-type experimental control studies (only 2 articles out of 75) that focus on loneliness and disability and their explanatory variables. This means that the data we have are so weak that they do not help us identify successful interventions based on scientific evidence. However, although it is true that our systematic review aimed to compile the main conclusions of the studies carried out on disability and loneliness, we have not obtained conclusive data on some questions that may arise in this area.

Specifically, we have not found any findings on whether loneliness in people with disability is a single construct or a collection of various subtypes or, in other words, whether we should consider the construct of loneliness in a unidimensional way. It may be necessary to carry out a comprehensive approach at various levels given the evidence of the lack of consensus for a single approach to loneliness among people with different types of disabilities. In line with this approach, in future research it would be very interesting to see how disability is being represented in mainstream media and becoming a new narrative (Casey and Stone, 2010). However, no article that meets the inclusion criteria for this systematic review includes social media as a study variable. Expanding on the multidimensionality of interventions in loneliness for people with disabilities also requires a deeper understanding of aspects such as the relationship between the degree of disability and the greater or lesser perception of loneliness, and knowing what psychological processes modulate the experience of loneliness, such as the perception of self-efficacy, competence, self-concept, or self-esteem.

We have not found any data on whether more disability leads to more perceived loneliness, or not. In a certain sense, it is logical to think that a greater degree of disability implies a greater perception of loneliness, but we have not found any work that has raised this working hypothesis and, consequently, that has confirmed it. Perhaps this finding is proof that non-disabled people assume that people with disabilities have a worse quality of life, but this idea contradicts what many people with disabilities actually think: “Non-disabled people assume disabled people have a low quality of life, which contradicts what people with disabilities experience” (Bogart, 2022). This gap between what non-disabled people believe and what people with disabilities actually experience is known as the disability paradox, which is a very interesting approach to this issue.

Likewise, we know that there are protective factors that reduce the perception of loneliness in people with a disability, such as having a job, or living in an environment without physical barriers. However, it is unknown whether these factors act in the same way across all types of disability. In other words, physical barriers may impose a greater perception of loneliness in people with physical disabilities, although it may not be a relevant factor in the case of intellectual disabilities. The articles analyzed do not provide insight into this issue.

Similarly, our systematic review does not provide conclusive data on whether the perception of loneliness may be linked to impaired self-regulation, especially with regard to executive functioning. Cognitive processes may modulate the experience of loneliness (Cacioppo and Hawkley, 2009), but we do not know which ones, nor to what extent. Although loneliness impairs executive functioning in part because it triggers implicit hypervigilance for social threats in the population without disabilities (Sin et al., 2021) we do not know if the same thing happens in people with disabilities. Finally, we have not found any relevant information on the most suitable types of assistance and support services either. As we pointed out earlier, we do not have any substantiated evidence that allows us to propose an intervention based entirely on these variables.

In terms of the interventions for people with disabilities, the same strategies have been adopted as for the non-disabled, including social skills training, enhanced social support, better opportunities for social interactions, and cognitive behavioral training. Is this a good approach to the problem? Does disability not require specific interventions, rather than adaptations of general interventions? For example, increasing social contact may be a good intervention strategy for people who have a strong social background (Masi et al., 2011). However, perhaps this is not the case for people who, because of their condition, see loneliness becoming unavoidable. And this could be the case for people with disabilities. Perhaps disability does not lead to loneliness, but instead it is the social structures that cause feelings of not belonging, and these lead to the perception of loneliness. Future research should go beyond the interventions that have been proposed, and offer interventions designed for and aimed at people with disabilities, but without forcing them on them.

As we have said above, disability has been presented as a factor that predisposes to perceived loneliness. However, after our work, we question whether disability leads directly to loneliness. It may not be a situation where the functional effect on people with disabilities leads them to a feeling of loneliness, but rather that it is the social structures that lead to feelings of not belonging, and this is what ultimately causes the loneliness. Disability is not what leads to or predisposes to loneliness. It is society that imprints loneliness on people with disability. Let’s change the starting point, and propose interventions not on those with disabilities, but on the society that surrounds them, the real key variable that inclines toward the real key variable that affects loneliness.

## Data availability statement

The raw data supporting the conclusions of this article will be made available by the authors, without undue reservation.

## Author contributions

BG-Z made a substantial contribution to the concept and design of the manuscript, led the systematic search of manuscripts, and was involved in drafting the manuscript. MP analyzed the manuscript included in the systematic review and involved in drafting the manuscript and revised it critically. MA was involved in drafting the manuscript and revised it critically and was the second reviewer to solve discrepancies between the other researchers. All authors contributed to the article and approved the submitted version.
