# Supplementary material for: Loneliness and disability: A systematic review of loneliness conceptualization and intervention strategies
Source: Front Psychol. 2023 Jan 25;13:1040651. doi: 10.3389/fpsyg.2022.1040651 (PMC9905422; doi:10.3389/fpsyg.2022.1040651)
Supplement: Supplementary file 1 [file Table_1.DOCX]

**Appendix 1**

| **Target population (2)** | **Description** | **Total ref.** | **%part.** |
| --- | --- | --- | --- |
| Children | up to age 12 | 3 | 4.00 |
| Children and adolescents | ages 12 and 13 | 4 | 5.33 |
| Adolescents | ages 13 to 19 | 11 | 14.67 |
| Adolescents and young people |  | 2 | 2.67 |
| Children, adolescents and young people |  | 2 | 2.67 |
| Adolescents, young people, and adults |  | 1 | 1.33 |
| Young people | age 20 | 2 | 2.67 |
| Young people and adults |  | 7 | 9.33 |
| Young people, adults, and the elderly |  | 10 | 13.33 |
| Adults | ages 30, 40, 50 | 9 | 12.00 |
| Adults and the elderly |  | 2 | 2.67 |
| The elderly | age +65 | 3 | 4.00 |
| The elderly and those dependent on others | ages 65+ and 80 | 6 | 8.00 |
| Most stages of the life cycle | adolescents, adults, the elderly, and those dependent on others | 1 | 1.33 |
| Average age (missing age range) |  | 6 | 8.00 |
| Missing information (no age information) | Missing age information | 6 | 8.00 |
| Blank | References not found | 10 |  |
|  | **Total** | **75** | **100.00** |
