# Supplementary material for: Loneliness and disability: A systematic review of loneliness conceptualization and intervention strategies
Source: Front Psychol. 2023 Jan 25;13:1040651. doi: 10.3389/fpsyg.2022.1040651 (PMC9905422; doi:10.3389/fpsyg.2022.1040651)
Supplement: Supplementary file 2 [file Table_2.DOCX]

**Appendix 2**

Characteristics of included studies

| **First author (year)** | **Country** | **Sample size (F/M)** | **Age** | **Type of disability** | **Intervention strategies** | **Area of knowledge** |
| --- | --- | --- | --- | --- | --- | --- |
| Alpass et al., 2003 | New Zealand | 217 (M) | Elderly people | - | - | General health care |
| Ballin et al., 2007 | Australia | 7 (F/M) | Adults | Physical (motor or not) | - | Assistance and support |
| Balto et al., 2019 | United States | 63 (F/M) | Adults | Various types | - | General health care |
| Beal et al., 2007 | United States | 659 (F) | Adults | Physical (motor or not) | - | Assistance and support |
| Bigby et al., 2017 | Australia | 34 (F/M) | Adults | Intellectual | - | Assistance and support |
| Bossaert et al., 2012 | Belgium | 216 (F/M) | - | Various types | - | Education |
| Broer et al., 2011 | Netherlands | - | - | Intellectual | Widening clients' social networks and making them feel less lonely through two different approaches: individualizing approach, and normalization. | Enabling environments |
| Burholt et al., 2017 | United Kingdom | 3.593 (F/M) | Elderly people | Cognitive impairment or Developmental Disorder | - | General health care |
| Chou et al., 2012 | United States | 101 (F/M) | - | Mental Disorder | - | Assistance and support |
| Cimarolli et al., 2018 | United States | 119 (F/M) | Elderly people | Various types | - | General health care |
| Cooper et al., 2009 | Australia | 6 (F/M) | Adults | Physical (motor or not) | - | Enabling environments |
| Dor et al., 2007 | Israel | 186 (F/M) | Adults | Mental Disorder | A segregative and an integrative psychosocial recreational program at the local community center. | Assistance and support |
| Eriksoon et al., 2012 | Norway | 17 | Adults | Mental Disorder | - | Assistance and support |
| Feldman et al., 2016 | Israel | 344 | - | Cognitive impairment or Developmental Disorder | - | Education |
| Fyffe et al., 2015 | Australia | 18 | Adults | Various types | A leisure Buddy Program that matched a community volunteer with an adult with intellectual disability, spending regular social and recreation time together. | Assistance and support |
| Gascon, 2009 | Australia | 55 (F/M) | Adults | Intellectual | - | Work and employment |
| Gilbert et al., 2013 | United States | 196 (F/M) | Adults | Various types | - | General health care |
| Heiman et al., 2015 | Israel | 482 (F/M) | Children, adolescents and young people | Cognitive impairment or Developmental Disorder | - | Education |
| Heiman, 2000 | Israel | 575 (F/M) | Children, adolescents and young people | Intellectual | - | Education |
| Heppe et al., 2020 | Netherlands | 316 | Children, adolescents and young people | Visual | - | Assistance and support |
| Hopps et al., 2001 | Canada | 24 (F/M) | Adults | Physical (motor or not) | - | General health care |
| Hopps et al., 2003 | Canada | 19 (F/M) | Adults | Physical (motor or not) | Involving both cognitive and behavioral techniques, which were tailored to each member of the group based on an in-person individual assessment. | Assistance and support |
| Howell et al., 2007 | United States | 82 (F/M) | Children, adolescents and young people | Various types | - | Education |
| Idan et al., 2014 | Israel | 856 (F/M) | Children, adolescents and young people | Cognitive impairment or Developmental Disorder | - | Education |
| Khazen et al., 2021 | United States | 69 (F/M) | Adults | Various types | - | General health care |
| Kotzer et al., 2007 | Israel | 374 (F/M) | - | Cognitive impairment or Developmental Disorder | A virtual supported intervention geared at training self-advocacy skills. | Education |
| Kruithof et al., 2018 | Netherlands | 19 (F/M) | Adults | Intellectual | A social work intervention that consisted of monthly three-course dinners that were organized in different districts of the city. | Assistance and support |
| Lehmann et al., 2013 | Netherlands | 667 | - | Intellectual | - | General health care |
| Lieb et al., 2017 | United States | 127 (F/M) | Children, adolescents and young people | Cognitive impairment or Developmental Disorder | - | Education |
| Lowe et al., 2021 | Ireland | 11 (F/M) | Adults | Physical (motor or not) | - | Enabling environments |
| Lui et al., 2020 | United States | 500 (F/M) | Adults | Physical (motor or not) | - | General health care |
| Lykke et al., 2019 | Denmark | - | - | - | - | Rehabilitation |
| Macdonald et al., 2018 | United Kingdom | 605 (F/M) | Adults | Various types | - | Assistance and support |
| Maddox et al., 2017 | United States | 25 (F/M) | Children, adolescents and young people | Cognitive impairment or Developmental Disorder | A Cognitive Behavioral Therapy (CBT) program designed to concurrently target anxiety and social impairment. | Education |
| Majorano et al., 2017 | Italy | 343 (F/M) | Children, adolescents and young people | Cognitive impairment or Developmental Disorder | - | Education |
| Mason et al., 2013 | United Kingdom | 11 (F/M) | Adults | Cognitive impairment or Developmental Disorder | - | Enabling environments |
| McVilly et al., 2006 | Australia | 51 (F/M) | All ages | Intellectual | - | Enabling environments |
| Ninan et al., 2013 | India | 30 | Children, adolescents and young people | Physical (motor or not) | - | Education |
| Nunkoosing, 2013 | United Kingdom | - | - | Cognitive impairment or Developmental Disorder | - | Enabling environments |
| Olsen, 2018 | United Kingdom | - | - | - | - | Enabling environments |
| Palmer et al., 2019 | United States | 240 (F/M) | Elderly people | Various types | - | General health care |
| Papoutsaki et al., 2013 | Greece | 154 (F/M) | Children, adolescents and young people | Various types | - | Education |
| Pavri et al., 2001 | United States | 30 (F/M) | Children, adolescents and young people | Cognitive impairment or Developmental Disorder | - | Education |
| Reversi et al., 2007 | Italy | 102 (F/M) | Children, adolescents and young people | Various types | - | Education |
| Rijken et al., 2008 | Netherlands | 1.265 (F/M) | Adults | Physical (motor or not) | - | Work and employment |
| Rijken et al., 2011 | Netherlands | 3.000 | Elderly people | Various types | - | General health care |
| Rojas-Pernia et al., 2020 | Spain | 23 | - | Intellectual | - | Enabling environments |
| Rosenstreich et al., 2015 | Israel | 335 (F/M) | Adults | Cognitive impairment or Developmental Disorder | A program with a single-session hope intervention workshop. | Education |
| Russell, 2009 | United States | 868 (F/M) | Adults | Physical (motor or not) | - | Assistance and support |
| Sharabi et al., 2011 | Israel | 887 (F/M) | Children, adolescents and young people | Cognitive impairment or Developmental Disorder | - | Education |
| Sharabi et al., 2016 | Israel | 178 (F) | - | Cognitive impairment or Developmental Disorder | - | Education |
| Smith, 2017 | United Kingdom | - | - | Physical (motor or not) | - | General health care |
| Stacey et al., 2013 | United Kingdom | 5 (M) | Adults | Cognitive impairment or Developmental Disorder | A narrative therapy based group approach, enabling participants to develop 'experience near' descriptions of loneliness and its effects, and to identify their abilities, strengths, and resources. | Education |
| Stancliffe et al., 2007 | United States | 1.002 (F/M) | Adults | Intellectual | - | Assistance and support |
| Stancliffe et al., 2009 | United States | 1.881 (F/M) | - | Various types | - | Assistance and support |
| Stancliffe et al., 2010 | United States | 7.996 (F/M) | Adults | Various types | - | Enabling environments |
| Stewart et al., 2011 | Canada | 22 (F/M) | Children, adolescents and young people | Physical (motor or not) | Computer-mediated peer support intervention program’. Topics included living independently, health concerns, bullying, making friends, career planning, traveling, sports, and building relationships. | Assistance and support |
| Stickley et al., 2017 | Japan | 7.403 (F/M) | - | Cognitive impairment or Developmental Disorder | - | General health care |
| Storch et al., 2009 | United States | 56 (M) | Children, adolescents and young people | Physical (motor or not) | - | General health care |
| Styron et al., 2006 | United States | 60 (F/M) | - | Mental Disorder | - | Assistance and support |
| Tamar et al, 2018 | United States | 91 | Adults | Physical (motor or not) | A 12-week educational socialization program with two types of groups: educational and social outing. The educational groups focused on teaching self-management strategies (e.g., thoughts, goals, plans, actions, etc.) for symptoms that prevent social interactions of any kind, especially social outings. | Education |
| Tilly, 2019 | United Kingdom | - | - | Cognitive impairment or Developmental Disorder | - | Enabling environments |
| Tough et al., 2018 | Switzerland | 246 (F/M) | - | Physical (motor or not) | - | Assistance and support |
| Tzonichaki et al., 2002 | Greece | 38 (F/M) | Adults | Physical (motor or not) | - | Rehabilitation |
| Van Asselt-Goverts et al., 2018 | Netherlands | 8 (F/M) | Adults | Intellectual | A semi-structured group training with group sessions. | Education |
| Van Hees et al., 2020 | Netherlands | 1.775 | Elderly people | Physical (motor or not) | - | Assistance and support |
| Vitman et al., 2018 | Israel | 13.828 (F/M) | Elderly people | - | - | Enabling environments |
| Warner et al., 2012 | United States | 1.500 (F/M) | Adults | - | - | Assistance and support |
| Warner et al., 2019 | United States | 138 (F) | Elderly people | Physical (motor or not) | - | General health care |
| White et al., 2009 | United States | 20 (F/M) | Children, adolescents and young people | Cognitive impairment or Developmental Disorder | - | General health care |
| Wilson et al., 2020 | Australia | 18 (M) | Children, adolescents and young people | Intellectual | A 6- month mentoring intervention to help counter barriers to employment. | Work and employment |
| Wormald et al., 2019 | Ireland | 317 (F/M) | - | Intellectual | - | General health care |
| Ylmaz et al., 2018 | Turkey | 56 (F/M) | Children, adolescents and young people | Cognitive impairment or Developmental Disorder | - | Education |
| Yu et al., 2005 | China | 98 | - | Cognitive impairment or Developmental Disorder | - | Education |
| Zahmacioglu et al., 2017 | Turkey | 117 (F/M) | Children, adolescents and young people | Cognitive impairment or Developmental Disorder | - | General health care |

**Key**

Age (mean)

| Children, adolescents and young people | Under 25 years old |
| --- | --- |
| Adults | 18 years old and above (includes all ages, i.e. older than 75 years old) |
| Elderly people | Over 65 years old (when focused specifically on this age group) |
| All ages | Includes all ages |
| - | No information available or we have an average age without reference to the minimum and maximum values |
